# Ancient Roman bacterium against current issues: strain Aquil_B6, *Paenisporosarcina quisquiliarum,* or *Psychrobacillus psychrodurans*?

**DOI:** 10.1128/spectrum.00686-23

**Published:** 2023-11-17

**Authors:** Andrea Colautti, Giuseppe Comi, Enrico Peterlunger, Lucilla Iacumin

**Affiliations:** 1 Department of Agricultural, Food, Environmental and Animal Science, University of Udine, Udine, Italy; Institute of Parasitology, Biology Centre, ASCR, Ceske Budejovice, Czechia

**Keywords:** *Psychrobacillus* spp., paleomicrobiology, *Paenisporosarcina* spp., ancient microbes, WGS

## Abstract

Thanks to the development of whole-genome sequencing technologies, nowadays researchers have access to rapid, effective, and precise resources for the genetic analysis of prokaryotes. The use of metrics that consider the entire DNA sequence, such as average nucleotide identity and digital DNA-DNA hybridization calculation, in fact, allows better results when compared to single-target gene analysis. However, it is increasingly important to employ reference databases containing correct information with high-quality sequences. The aim of this work is to taxonomically identify an unknown bacterial strain isolated from an ancient Roman amphora. However, it was necessary to resequence the genomes of the reference strains *Paenisporosarcina quisquiliarum* SK 55 and *Psychrobacillus psychrodurans* DSM 11713 to obtain correct and more reliable sequences than those available. It was, therefore, possible to uniquely identify the strain Aquil_B6 as *Psy. psychrodurans*. Also, to obtain further genetic information, given the low number of sequences available for this species, the genome of strain DSM 30747 was sequenced. Further characterization was made to identify the presence of possible genetic characteristic features such as CRISPR-Cas, prophages, resistance factors, and bacteriocins of this species, making a comparison between the available genomes of *Paenisporosarcina quisquiliarum* SK 55 and *Psychrobacillus psychrodurans* strains DSM 11713, DSM 30747, and Aquil_B6, to evaluate the impact of time on the evolution of the considered species.

**IMPORTANCE:**

Since 1988, through the United States government’s founding, the National Center for Biotechnology Information (NCBI) has provided an invaluable service to scientific advancement. The universality and total freedom of use if on the one hand allow the use of this database on a global level by all researchers for their valuable work, on the other hand, it has the disadvantage of making it difficult to check the correctness of all the materials present. It is, therefore, of fundamental importance for the correctness and ethics of research to improve the databases at our disposal, identifying and amending the critical issues. This work aims to provide the scientific community with a new sequence for the type strain *Paenisporosarcina quisquiliarum* SK 55 and broaden the knowledge of the *Psychrobacillus psychrodurans* species, in particular, considering the ancient strain Aquil_B6 found in an ancient Roman amphora.

## INTRODUCTION

One of the most important and necessary practices in microbiology is the taxonomic identification of unknown bacterial strains. The 16S rRNA gene sequencing technique is one of the most commonly used identification methods that, however, shows several limitations. For example, for many phylogenetically close species, the potential of the 16S rRNA gene to provide taxonomic resolution at the species level is inadequate, being not able to uniquely and certainly identify the species of correspondence. This has been reported for the former genus *Bacillus* spp., where three reference strains shared greater than 99.5% similarity among the 16S rRNA gene sequence (1). A great similarity of this DNA region also characterizes the recently diverged species, thus making the distinction of several microbial species ineffective (2, 3). The quality of the sequences deposited in databases also plays a significant role in the results obtained. However, the reported large amount of low-quality sequences deposited in the past years, as well as the estimated presence of errors or chimeras, may pose additional challenges in the identification procedures (4). Alternatively, the sequencing of other target genes, while capable of resolving some of these issues, is more time-consuming. Nowadays, whole-genome sequencing (WGS), thanks to its greater accessibility, is one of the most reliable and effective techniques for obtaining a unique and valid identification as well as complete knowledge of the genetic characteristics of the investigated bacteria. WGS overcomes the issues associated with 16S rRNA gene sequencing due to the availability of methods based on the comparison of the entire DNA sequence that have been developed over time for more precise results. Since 1960, one of the most effective bacterial identification techniques has been DNA-DNA hybridization (DDH), which is efficient in providing stable and reproducible results. However, due to the complexity of its execution, it has been gradually supplanted by newly developed methods of comparison since the advent of the genomics era. These include the average nucleotide identity (ANI), a metric based on the level of genomic similarity between the coding regions of two genomes (5). In addition to ANI, the increasing affordability of genomic sequences has enabled the calculation of DDH *in silico* via the measurement of digital DNA-DNA hybridization (dDDH) (6). This made it possible to replace the complex laboratory operations necessary for the evaluation of DDH with simple and user-friendly interface programs, such as the free tool Genome-to-Genome Distance Calculator (GGDC) (7, 8). Based on this tool, the Type Strain Genome Server (TYGS) was developed, which can identify a query strain based on its entire genetic sequence by comparing dDDH values against an updated database of prokaryotic genomes of reference strains (9). However, even when using these tools, the quality of the databases used is critical. In fact, due to inconsistencies in the genetic sequences of some reference strains, using TYGS, it was not possible to taxonomically identify a bacterial strain sequenced in a previous work (10). The unknown bacterial strain Aquil_B6, isolated together with seven other bacilli from the content of an ancient Roman amphora of the 4th and 5th centuries AD, clustered for dDDH values both with the available reference sequences for *Psychrobacillus psychrodurans* DSM 11713 and *Paenisporosarcina quisquiliarum* SK 55. The sequences of these two strains, whose WGS projects are deposited on NCBI under the accession numbers GCA_900109875 and GCA_900114885, were reported as “anomalous assembly”. Furthermore, TYGS showed that comparing these two sequences, both reference strains could be identified as the same species. To resolve this issue and obtain new trustworthy genome sequences to be deposited on the NCBI database, for correctly and uniquely classifying strain Aquil_B6, the DNAs of both strains were resequenced. Due to the limited availability of other *Psy. psychrodurans* genomes, the sequencing of the *Psy. psychrodurans* DSM 30747 strain was also performed to provide the scientific community with new information on this species. Therefore, the aim of this work was to correctly identify strain Aquil_B6, after understanding whether the deposited sequences were correct. In fact, if the resequencing confirms the correctness of the two reference sequences, it would suggest to consider the possibility of merging the two species; on the contrary, if the presence of errors was ascertained, the separation of the two species as reported in the literature would be confirmed (11, 12), consequently obtaining a unique identification of the Aquil_B6 strain.

## RESULTS AND DISCUSSION

### Analysis of the available genomes on NCBI database of the species *Psy. psychrodurans* and *Pae. quisquiliarum*

**Fig 1:**
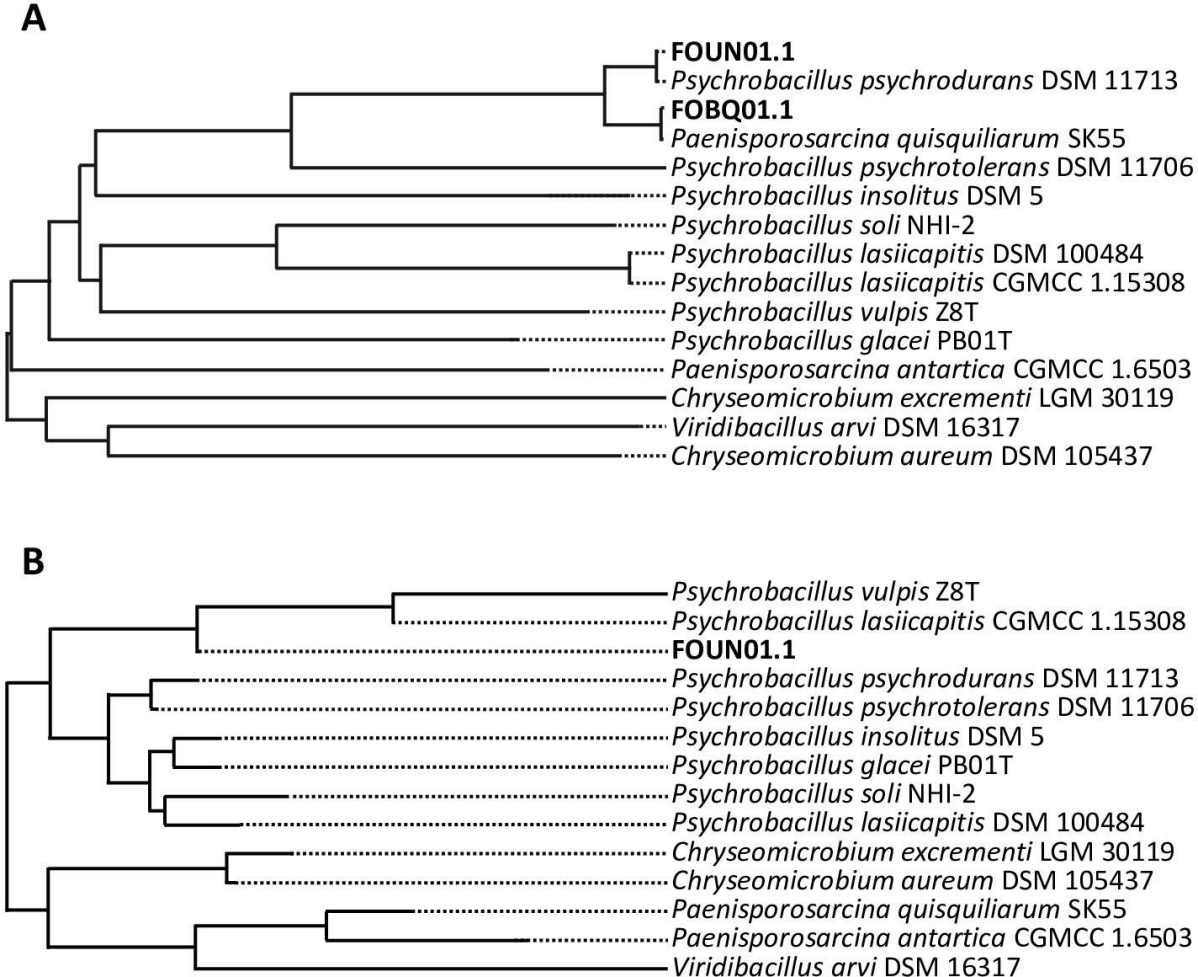
TYGS clusterization of FOUN01 and FOBQ01 sequences, considering (**A**) WGS and (**B**) 16S rRNA gene sequences. In panel B, sequence FOBQ01 is not present as it did not match with any reference sequence in the TYGS database.

During the preliminary identification of strain Aquil_B6 performed in a previous work (10), although a greater similarity with the reference strain *Psy. psychrodurans* DSM11713 (dDDH = 88.8%) emerged, a match with dDDH values >70% also emerged with the reference strain *Pae. quisquiliarum* SK 55. It was, therefore, decided to deepen this inconsistency in detail by verifying the correctness of the sequences deposited for these two reference strains. In the following figures and tables, the genomes of *Psy. psychrodurans* DSM11713 (assembly accession GCA_900114885, WGS project FOUN01) and *Pae. quisquiliarum* SK 55 (assembly accession GCA_900109875, WGS project FOBQ01) already present in the TYGS database will be differentiated from the newly sequenced genomes by the indication *Old* (O) after their name. These genome sequences downloaded from NCBI were used as query sequences to obtain whole-genome clustering (Fig. 1A), resulting in dDDH values reported in Table 1. As expected, the query sequences matched with the reference strains; however, FOUN01 and FOBQ01 genome sequences shared a dDDH value of 80% with a 0.04% difference in %GC. This dDDH value is above the threshold reported in the literature (dDDH=70) to consider two strains as separate species, suggesting that they belong to a single species (5, 13 – 15). A further contradictory result was provided from the clustering based on the 16S rRNA gene sequence in Fig. 1B, where the FOBQ01 sequence did not match with any deposited 16S rRNA gene sequence, and without showing any match with the 16S rRNA gene sequence of *Pae. quisquiliarum* SK 55 deposited with accession number DQ333897. These findings contradicted the taxonomic descriptions of these two distinct species (11), and the absence of a match with the 16S rRNA gene sequence for *Pae. quisquiliarum* SK 55 suggested that the reference sequence (named FOBQ01 WGS project) used for the type strain *Pae. quisquiliarum* by TYGS contained sequencing errors.

**TABLE 1:** dDDH calculation of FOUN01 and FOBQ01 sequences downloaded from NCBI through TYGS

Query strain (NCBI)	Subject strain (TYGS)	dDDH (d_4_, in %)	C.I. (d_4_, in %)	G + C content difference (%)
FOUN01	*Psychrobacillus psychrodurans* DSM 11713 (O)	100.0	[100.0–100.0]	0.0
FOBQ01	*Paenisporosarcina quisquiliarum* SK 55 (O)	100.0	[100.0–100.0]	0.0
FOUN01	*Paenisporosarcina quisquiliarum* SK 55 (O)	80.0	[77.0–82.6]	0.04
FOBQ01	*Psychrobacillus psychrodurans* DSM 11713 (O)	80.0	[77.0–82.6]	0.04
FOBQ01	FOUN01	80.0	[77.0–82.6]	0.04

### Genetic features of newly sequenced strains

The accession numbers for the sequence read archive (SRA) and WGS of the newly assembled genomes analyzed in this study contained in bioproject PRJNA840842, as well as the previous *Psy. psychrodurans* Aquil_B6 from bioproject PRJNA811801, together with the total length and %GC comparison with previously available homologous strain sequences are reported in Table 2. It can be noted that *Psy. psychrodurans* DSM11713 and DSM30747 genomes were characterized by a similar length (4.03 and 4.06 Kbp, respectively) and %GC content (36.01%–36.05%). When compared to the previously sequenced *Psy. psychrodurans* DSM11713 (O) genome, the new assembly showed a close length and %GC, suggesting the correctness of both sequencings. The genome of strain Aquil_B6 resulted very close to the reference strain although it differed by a slightly longer length (4.26 Kbp) and a lower GC percentage (35.94%). The new genome assembly of *Pae. quisquiliarum* SK 55, on the other hand, was much shorter (3.14 Kbp) and had a higher percentage of GC (39.71%) with respect to the deposited old sequence, which appeared to be in contrast with the obtained results, showing a longer length of 4.03 Kbp (difference of 893,465 bp) and a %GC difference of 3.75%. Therefore, the non-correspondence of the two strains under consideration is demonstrated by these values. Thus, these results confirmed that the old sequence of *Pae. quisquiliarum* SK 55 (assembly accession GCA_900109875, WGS project FOBQ01 FOBQ01) was incorrect and responsible for the doubtful identification of strain Aquil_B6.

**TABLE 2:** General features and accession numbers

Assembly	Total length	GC%	WGS accession	SRA accession
Newly assembled strains				
DSM11713	4027030	36.01	JAMKBK000000000	SRR19330377
DSM30747	4064800	36.05	JAMKBI000000000	SRR19330375
Aquil_B6	4256356	35.94	JAKXDZ000000000	SRR18190499
SK55	3140025	39.71	JAMKBJ000000000	SRR19330376
Previous reference strains				
SK55 (O)	4033490	35.96	FOBQ01000000	-
DSM11713 (O)	4016876	36.00	FOUN01000000	-

The assembly parameters and genetic characteristics of the strains under analysis are reported in Table 3. All genomes were assembled with L50 values between 5 and 6, with completeness values above 99.34% confirming the good results of the sequencing process. In addition to the differences in total length and %GC already analyzed above, the strains belonging to the *Psy. psychrodurans* species showed a greater number of genes, in the range of 3,987 (for strain DSM 30747) and 4,295 (for strain Aquil_B6), compared to *Pae. quisquiliarum* SK 55, which presented 3,203 genes.

**TABLE 3:** Assembly statistics of the new assemblies

Assembly	Contigs	N50	L50	Completeness (%)	CDS	Gene	tRNA	tmRNA
DSM11713	57	232522	5	100	3945	4018	72	1
DSM30747	45	222761	6	99.34	3916	3987	70	1
Aquil_B6	79	253084	6	100	4224	4295	70	1
SK55	39	214672	5	99.34	3131	3203	71	1

### Taxonomic analysis of new strains

**Fig 2:**
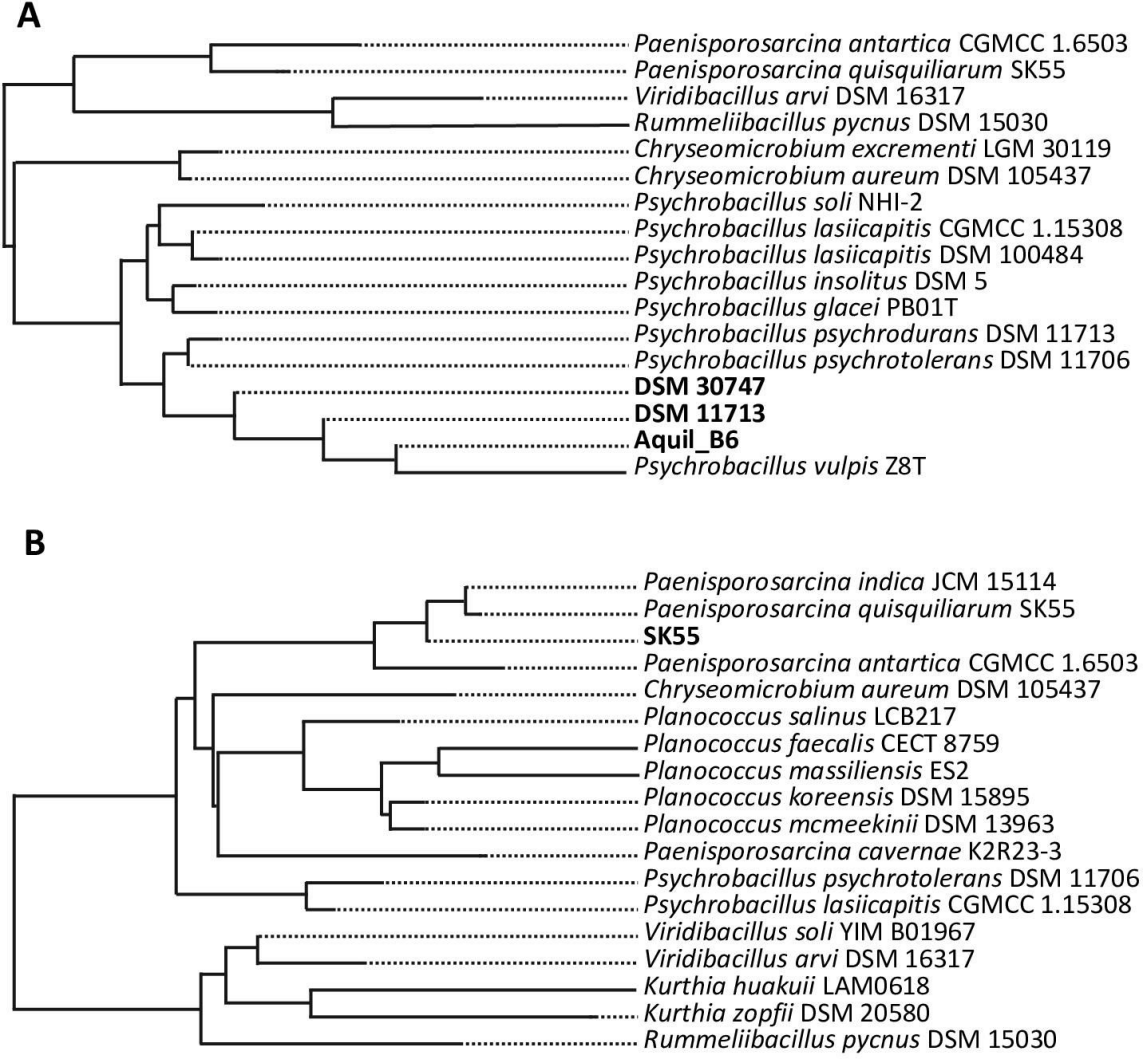
16S rRNA gene sequence clustering of *Psy. psychrodurans* DSM 30747, DSM 11713, and Aquil_B6 (**A**) and *Pae. quisquiliarum* SK 55 (**B**).

Using the 16S rRNA gene sequence prediction function implemented in the TYGS tool, a first clustering analysis conducted with only this portion of the genome revealed that the three strains of *Psy. psychrodurans* DSM 11713, DSM30747, and Aquil_B6, although grouped together, did not show a perfect match with the 16S rRNA gene sequence deposited with the accession number AJ277984 of *Psy. psychrodurans* DSM 11713, showing, on the contrary, a higher affinity with *Psy. psychrotolerans* DSM 11706 and *Psychrobacillus vulpis* Z8T (Fig. 2A). Once again this result suggests that the only comparison of 16S rRNA gene sequences between closely related species can lead to inaccurate identifications. Vice versa, the 16S rRNA gene sequence analysis of *Pae. quisquiliarum* SK 55 correctly identified the strain by clustering directly with the homologous reference deposited with the accession number DQ333897 (Fig. 2B).

**Fig 3:**
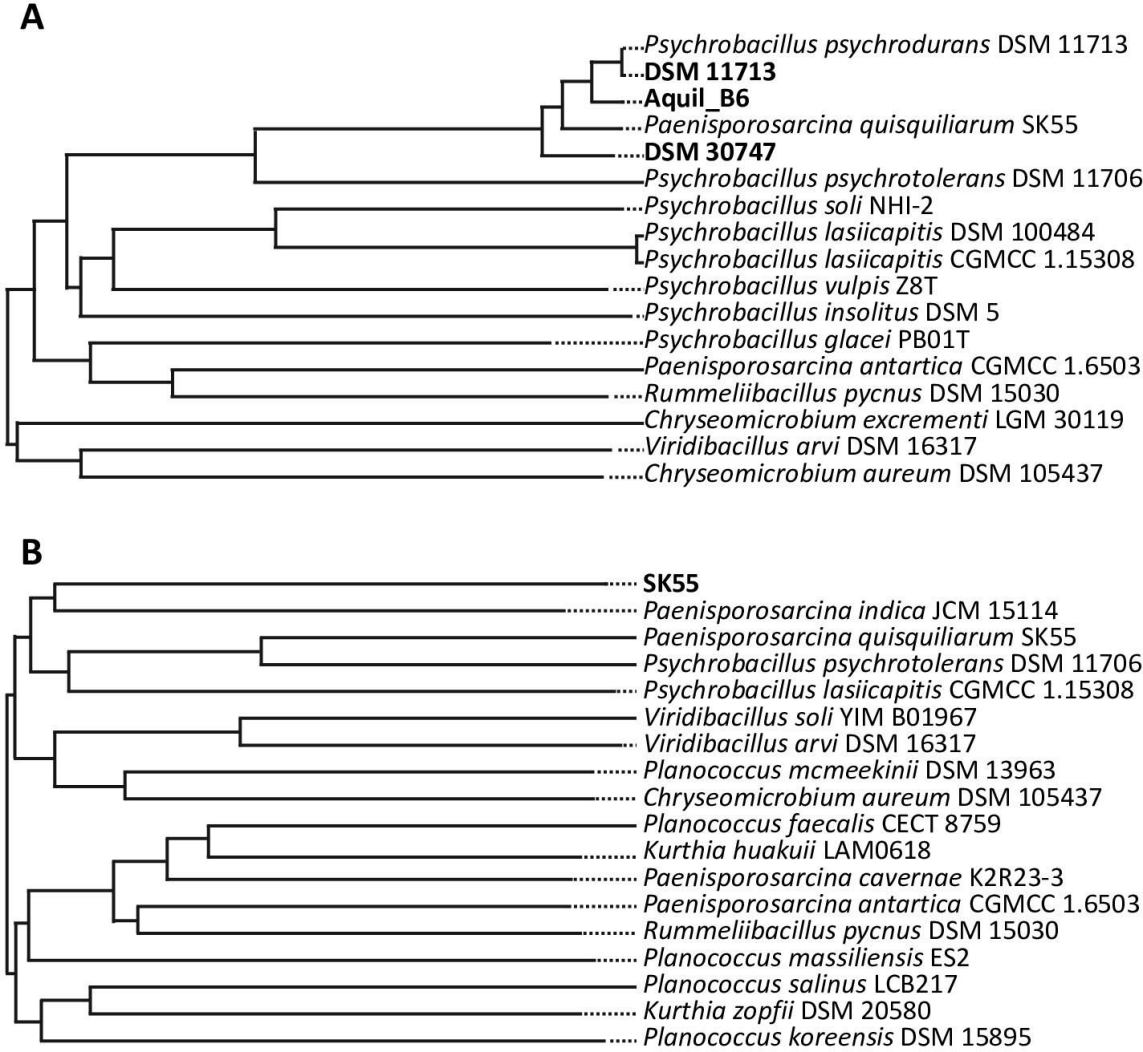
WGS clustering of *Psy. psychrodurans* DSM 30747, DSM 11713, and Aquil_B6 (**A**) and *Pae. quisquiliarum* SK 55 (**B**).

Using the calculation of dDDH value on the whole-genome sequence as a comparison parameter, strains DSM 11713, DSM 30747, and Aquil_B6 clustered with the sequence FOUN01 of the reference strain *Psy. psychrodurans* DSM 11713, however, together with the incorrect *Pae. quisquiliarum* SK 55 sequence (Fig. 3A). The two assemblies of strain DSM 11713 had a dDDH value of 100% based on the dDDH values reported in Table 4, confirming the accuracy of both sequences. Strains DSM 30747 and Aquil_B6 were also correctly attributed to this species, with dDDH values higher than the threshold limit, corresponding to 72% and 89%, respectively. It was, therefore, possible to identify with certainty the Aquil_B6 strain as belonging to the species *Psy. psychrodurans*. On the contrary, the whole-genome analysis of our new *Pae. quisquiliarum* SK 55 sequence, resulted in a “new species” as observable in Table 5, with no matches above the dDDH threshold limit. The most similar species *Rummeliibacillus pycnus* DSM 15030 had dDDH similarity values of 24%, while the previous sequence deposited for SK55 had a dDDH similarity of 20% with a %GC difference of 3.75. The same considerations can be derived from the observation of the tree proposed in Fig. 3B where our sequence of the *Pae. quisquiliarum* SK 55 strain did not seem to have some correspondence with any of the proposed reference strains. The same conclusions can be drawn from the Taxonomy Check made by NCBI on the assemblage (ASM2756331v1), where the closest species resulted in *Paenisporosarcina indica* GCA_001939075.1 with an ANI similarity equal to 81.88%. These results obtained with the new sequence also confirmed the differentiation of the *Pae. quisquiliarum* species using the SK55 strain as a reference strain, as reported in the literature (12). This allowed us to resolve the issue and perform a correct classification, and this once again underlines the importance of verifying the deposited data sequences, and the necessity of the scientific community to start a discussion about this issue. In fact, nowadays, the increasing number of genomic and metagenomic material that is deposited presents in some cases a low quality or false species attribution. This causes problems and is detrimental to the quality of research and could lead to an exponential propagation of errors over time, compromising the results of scientific works.

**TABLE 4:** dDDH values for *Psy. psychrodurans* strains

	Aquil_B6	DSM11713	DSM30747	DSM 11713 (O)	SK 55 (O)	
Aquil_B6	-	0.07	0.11	0.05	0.01	G + C content difference (%)
DSM11713	88.8	-	0.04	0.01	0.05	
DSM30747	71.9	71.9	-	0.05	0.09	
DSM 11713 (O)	88.8	100.0	71.8	-	0.04	
SK 55 (O)	79.0	80.0	73.6	80.0	-	
	dDDH (d_4_, in %)					

**TABLE 5:** dDDH values for *Pae. quisquiliarum* SK 55

Query strain	Subject strain	dDDH (d_4_, in %)	C.I. (d_4_, in %)	G + C content difference (in %)
“SK55”	*Paenisporosarcina quisquiliarum* SK 55 (O)	20.8	[18.6–23.2]	3.75
“SK55”	*Paenisporosarcina indica* JCM 15114	21.8	[19.6–24.3]	1.23
“SK55”	*Paenisporosarcina cavernae* K2R23-3	22.5	[20.3–25.0]	0.08
“SK55”	*Paenisporosarcina antarctica* CGMCC 1.6503	22.8	[20.5–25.2]	2.75
“SK55”	*Rummeliibacillus pycnus* DSM 15030	24.0	[21.7–26.5]	5.06

### Strain characterization

**Fig 4:**
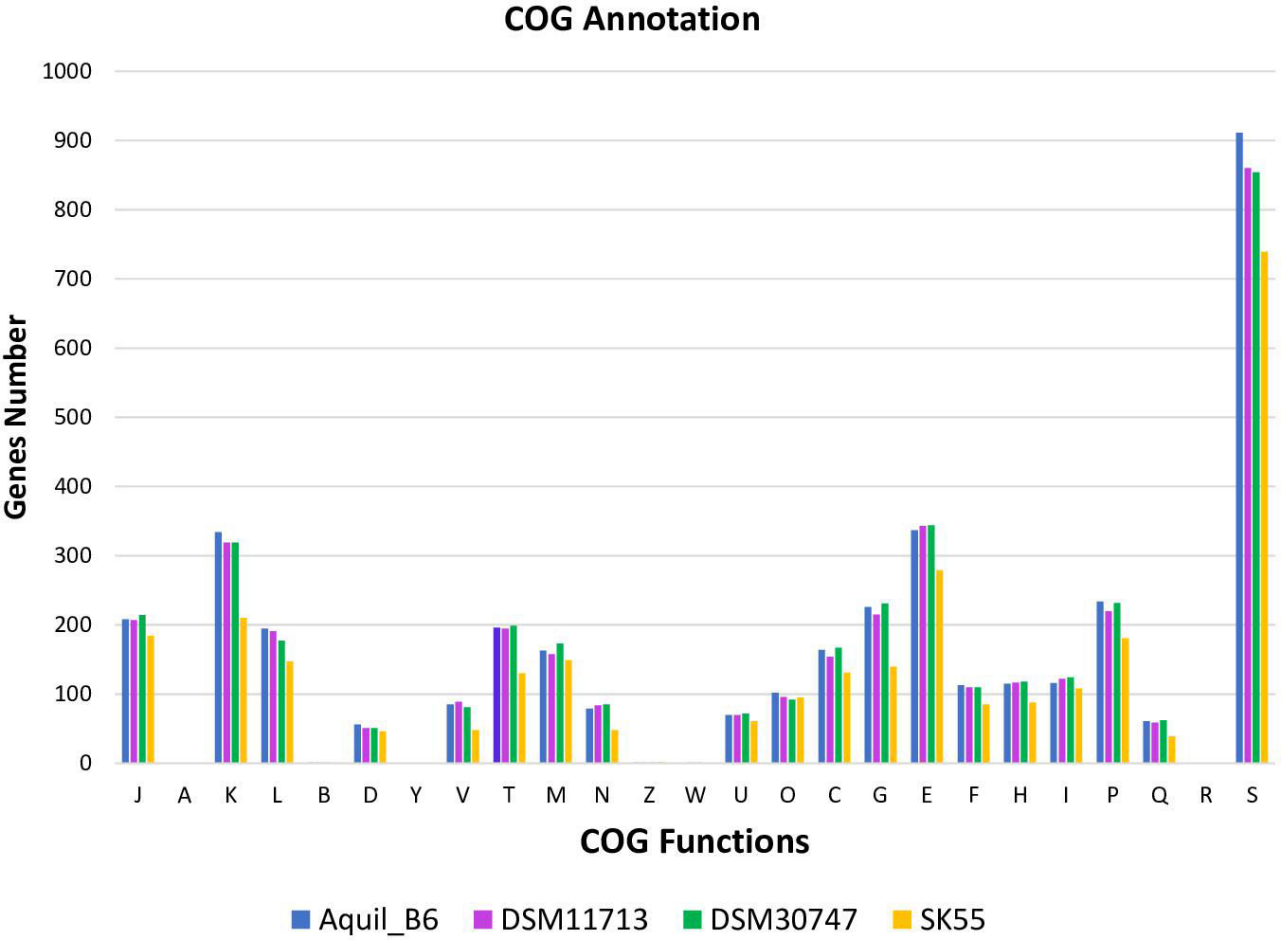
COG gene distribution.

Nowadays, more and more species belonging to the genus *Psycrobacillus* are being studied for various peculiar characteristics, including the ability to degrade oils (16), produce bio-emulsifiers (17), and phosphate-solubilizing ability (18). These capabilities are also associated with the ubiquitous discovery of this genus, ranging from Egypt (19) to polar ices (17, 20), from ancient findings (10) to cleanrooms of space observatories (as reported for the DSM 30,747 strain) or in clean-room environments of NASA (as reported in the bioproject PRJNA832800). The same considerations can be made for the genus *Paenisporosarcina*, an environmental bacterium of which many species were isolated in extreme environments (21). Given the small number of case studies on these species carried out, especially in the last period, it is important to continue their studies as their ability to resist adverse conditions could conceal important technological or industrial applications thanks to possible new metabolisms yet to be discovered (22 – 25). The COG annotation in Fig. 4 shows how genes were distributed based on the attributed function, which was identified in 76.05% of cases. Most genes (36.62%) were linked to metabolic functions, in particular, of amino acid transport and metabolism (E, 9.27%) followed by ion (P, 6.17%), carbohydrate (G, 5.78%), energy (C, 4.38%), lipid (I, 3.35%), coenzyme (H, 3.12%), nucleotide (F, 2.98%), and secondary metabolites (Q, 1.57%) metabolisms. The remaining 20.15% were allocated to cellular processes and signaling, the most important of which were signal transduction mechanisms (T, 5.12%) and cell biogenesis (M, 4.57%). Finally, 19.28% of the functions were assigned to information storage and processing, mainly in transcription (K, 8.41%), translation (J, 5.79%), and replication recombination and repair (L, 5.05%). It can be observed that between the different strains, a constant relationship of the different functions is maintained without significant variations.

Using the KEGG mapper, it was possible to reconstruct the metabolic pathways of the individual strains (Table S1). All three analyzed *Psy. psychrodurans* strains shared the same complete pathways, with two exceptions: (i) first, the metabolism of carbohydrates, in particular, strains Aquil_B6 and DSM 30747 showed the ability to synthesize UDP-galactose starting from galactose that was not predicted in the genome of the DSM 11713 strain and (ii) regarding the polyketide biosynthesis, only strain DSM 30747 had a fully reconstructed dTDP-L-rhamnose biosynthesis pathway. *Pae. quisquiliarum* SK55 distinguished from *Psy. psychrodurans* strains due to the absence of several metabolisms. In fact, considering the metabolism of sugars, the Leloir pathway for the degradation of galactose, the biosynthesis capability of glycogen from glucose-1P, and UDP-galactose from galactose were lacking. Also, in energy metabolism, no F-type ATPase was identified, and in the nucleotide, the metabolism for pyrimidine deoxyribonucleotide biosynthesis was missing. Moreover, the metabolism of amino acids presented incomplete pathways for the biosynthesis of threonine, methionine, valine/isoleucine, and ornithine, and in cofactors and vitamins, the metabolism was devoid of the thiamine salvage pathway, the biosynthesis of molybdenum cofactor, and a pathway for pyridoxal P and pantenoate biosynthesis. On the other hand, a complete pathway for formaldehyde assimilation was observed. Deoxyribonucleotide, lysine, and NAD biosynthesis as well as C1 unit interconversion and C5 isoprenoid biosynthesis capability were found. Of particular attention, the possible presence of a VraFG transporter resistance factor associated with antimicrobial peptides was underlined. Other features of the genomes were analyzed using specific tools described in the Materials and Methods section. Through functional annotation performed with Prokka, the possible presence of various bacterial cold-shock proteins, which confer resistance to low temperatures, was also identified (26). In all strains under examination, the presence of CspA, CspB, CspC, and CspLA was in fact predicted. The presence of possible resistance factors or bacteriocins was not detected using RGI and BAGEL4. The presence of prophages predicted using PHASTER revealed the presence of a prophage only in the genome of the strain DSM11713, which was fully identified as Paenibacillus phage PG1 (NC_021558). Also, using CRISPRCasFinder, no possible CRISPR-Cas defense systems were predicted. Considering the fact that in some cases the literature reports that prophages and cryptic prophages can represent up to a fifth of the genetic material in bacteria (27), and it is possible to hypothesize that such a low presence of identified prophages may be attributable to the difficulty of identification by part of the bioinformatics tools currently available (28), in addition to the not yet reached completeness of the related databases. Deeper and wider future knowledge on prophages and CRISPR-Cas systems could come in handy in phylogenetic studies and be a powerful tool for reconstructing the evolution and evaluating the differences of prokaryotes, since prophages, in addition to directly influencing bacterial evolution (29), can provide valuable information on the biological history of the bacterium (28, 30). No strict matches were found in the research on possible resistance factors to biocides and heavy metals conducted using BacMet database. Only two possible resistance factors with an identity percentage greater than 80% were identified for strain Aquil_B6 (WP_063593029 nitrite reductase and WP_063593260 heavy metal translocating P-type ATPase).

### Relation between *Psy. psychrodurans* strains

ANI values also confirmed the results of the dDDH analysis among the *Psy. psychrodurans* strains examined (Table 6). Despite the major differences in genome length and %GC, the reference strain DSM 11713 and the Aquil_B6 strain shared a dDDH similarity of 88.8% and an ANI of 98.62%, putting them closer together than the DSM 30747 strain, which had a dDDH value of 71.9% and ANI of 96.69% when compared to the reference strain.

**TABLE 6:** ANI and dDDH matrix for *Psy. psychrodurans* strains

		dDDH		
		Aquil_B6	DSM 11713	DSM 30747
ANI	Aquil_B6	-	88.8	71.9
	DSM 11713	98.629	-	71.9
	DSM 30747	96.65161	96.68996	-

**Fig 5:**
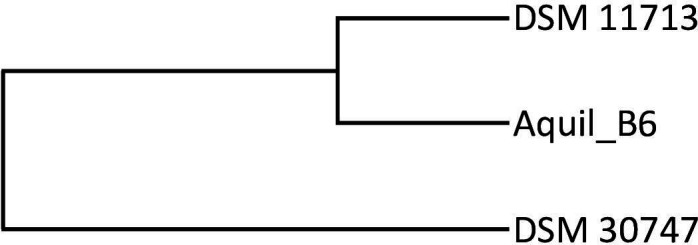
Phylonium UPGMA evolutionary distance tree.

Similar results were obtained by estimating the evolutionary distances of the genomes, and also in this case, the strains DSM 11713 and Aquil_B6 were evolutionarily more similar at a temporal level than the strain DSM 30747, which diverges more markedly (Fig. 5).

**Fig 6:**
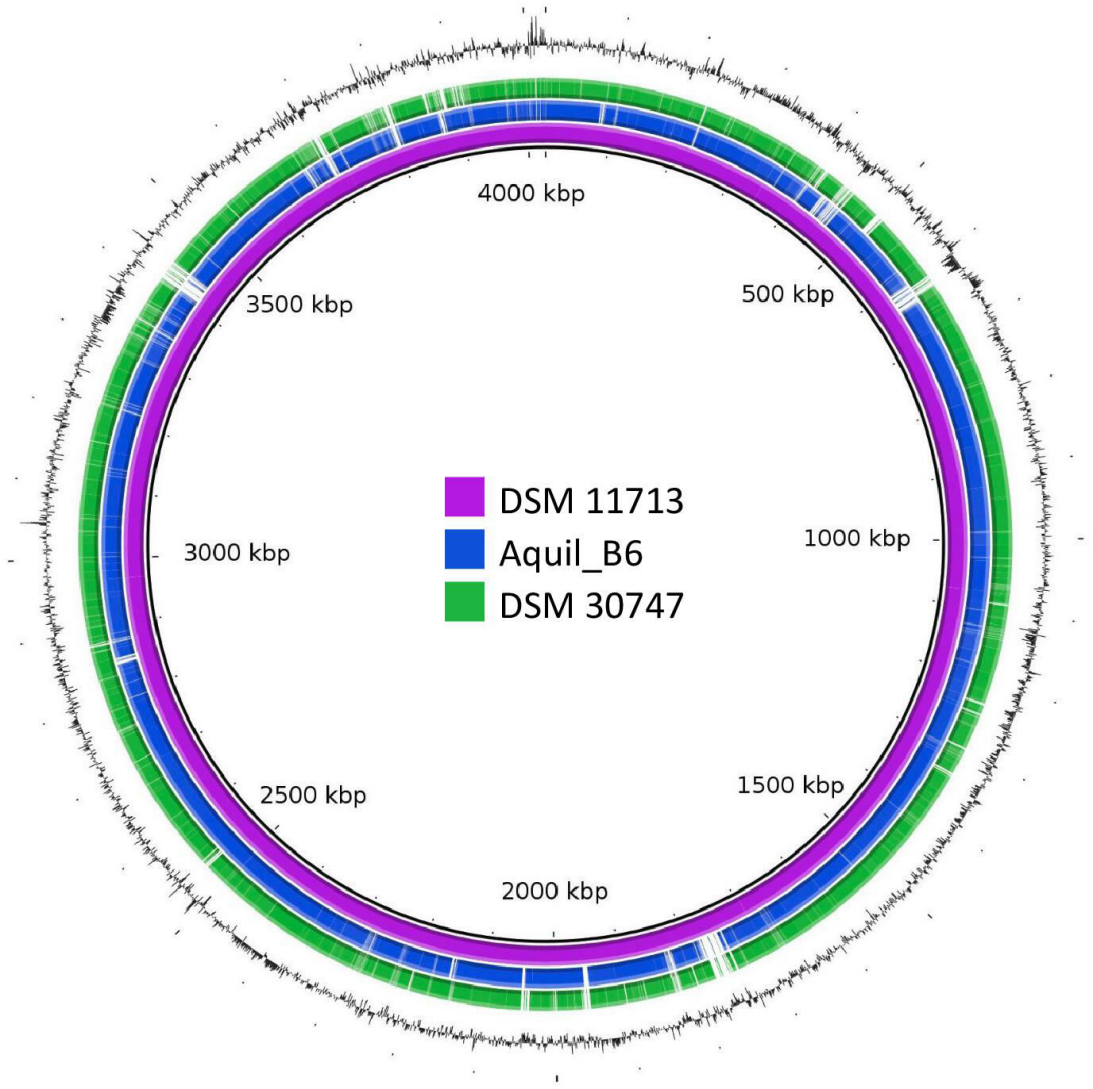
Genome comparison obtained by BRIG of *Psy. psychrodurans* strains (circular graph).

A first comparative genome analysis of *Psy. psychrodurans* was shown in Fig. 6. This assessment obtained by BRIG, in addition, to the %GC representation, compared the genomes of strains Aquil_B6 and DSM 30747 to the reference strain DSM 11713 with a threshold value of 50%. It was possible to observe several points of differentiation between the reference and query genomes, also highlighting the several differences between strains Aquil_B6 and DSM 30747.

**Fig 7:**
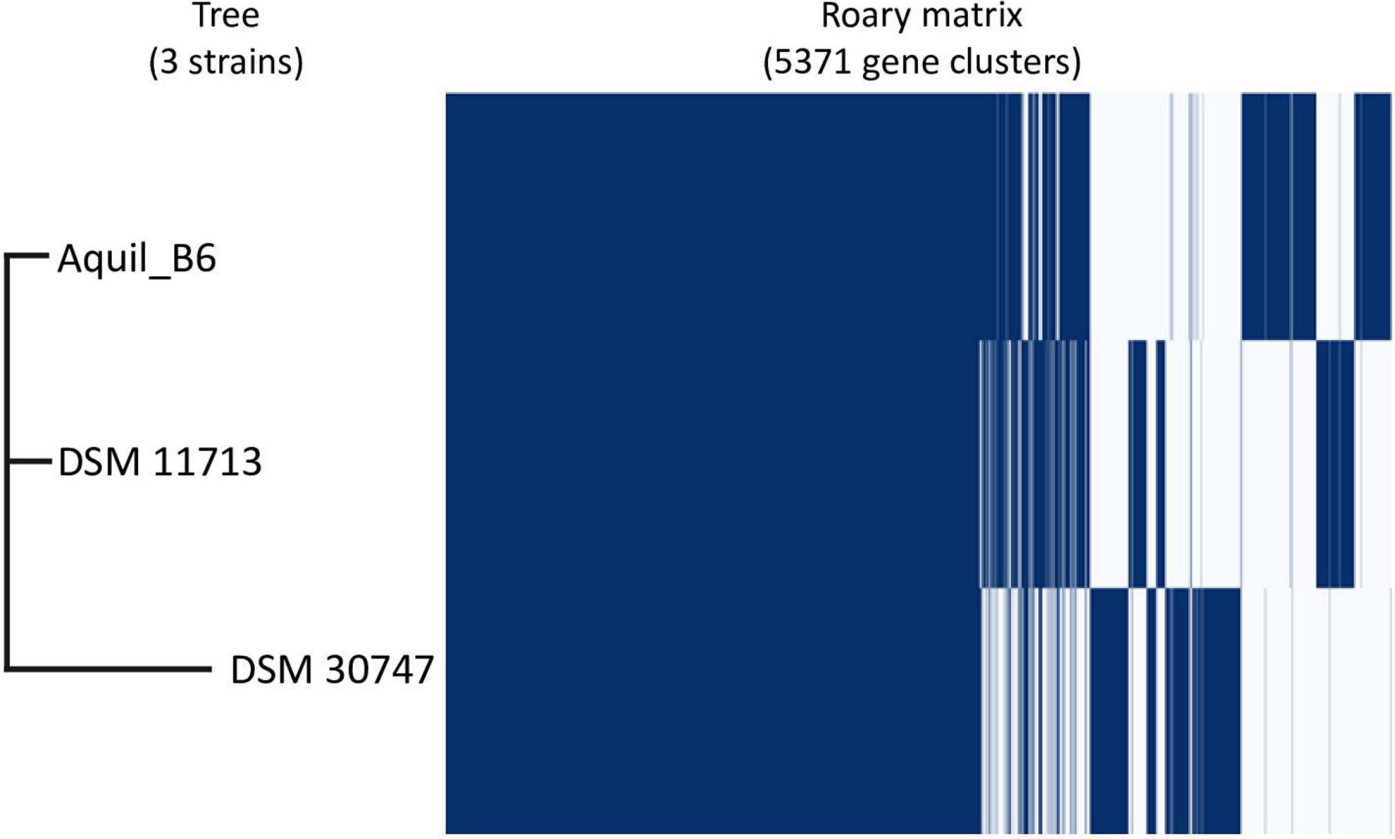
Roary gene matrix for *Psy. psychrodurans* strains.

The higher correlation between strains Aquil_B6 and DSM11713 was also highlighted by the analysis of the pangenome made with Roary, as reported in Fig. 7, which depicted the distribution of genes in the different genomes. Of the total of 5,371 genes, a core genome of 3,045 genes shared among all three genomes and a shell genome shared from at least two genomes composed of 624 genes were identified. Strain Aquil_B6 was found to share a greater number of genes with strain DSM11713 (3,467 genes) than with strain DSM 30747 (3137 genes). From the presence/absence table also obtained through Roary, 640 genes were present exclusively in the strain. The analysis of the pangenome, reported in the literature as a valid method of taxonomic classification (31 – 33), once again confirmed the belonging of these three strains to the same species. The high number of shared genes compared to the unique genes of each strain testifies to the close connection between these strains, whose genomes are the only ones currently available in the literature for comparison, thus laying the basis for future more in-depth research on this species.

### Conclusions

Through the resequencing of the genomes of *Psy. psychrodurans* DSM 11713 and *Pae. quisquiliarum* SK 55, it was possible to identify issues in the genomic sequence deposited for the latter. A new corrected reference genome for this species has, therefore, been provided. In the light of these results, it was possible to confirm the belonging of the strain Aquil_B6 to the *Psy. psychrodurans* species. The discovery of this microorganism in the internal content of a Roman amphora, which remained sealed for a long time, confirms once again the resistance of this species to difficult environmental conditions as reported in the literature. The strain DSM 11713 was genetically the most similar to the strain Aquil_B6; however, given the small number of genomes available, all sequenced in this work, it was not possible to make an effective comparison on the possible differences that such a prolonged isolation has produced on the strain. Anyway, fundamental steps have been taken on the knowledge of these species, which in the future can be expanded to understand the genetic basis of the resistance characteristics of these environmental organisms.

## MATERIALS AND METHODS

### Description of the amphora and its contents

The Aquil_B6 strain, as reported in detail in a previous work (10), was isolated from an ancient Roman amphora, dated between the 4th and 5th centuries AD based on the construction technique (Byzacene amphora of African origin) and site of the excavations (34). The amphora was discovered in Aquileia (UD, Italy) and was still intact and sealed with a cementitious compound, thus preventing microbial contamination. The amphora was opened in sterility, and the contained dehydrated material, among which it was possible to distinguish grape berries and seeds, dehydrated leaves, as well as a resinous compound, was collected in sterility for microbiological analyses. The material found inside suggests that the original content of the amphora could have been wine; however, more in-depth studies are ongoing.

### Bacterial strains and culture conditions

Strain Aquil_B6 was isolated as previously described from a Roman amphora. It was stored at −80°C at the University of Udine in brain heart infusion (BHI) broth (Oxoid, Germany) added with 20% glycerol (Sigma, Germany). The other strains used in this study were obtained directly from the corresponding collections of microorganisms in freeze-dried form: *Psy. psychrodurans* DSM 11713 and DSM 30747 *s*trains were obtained from the Leibniz Institute DSMZ—Deutsche Sammlung von Mikroorganismen und Zellkulturen Collection (Germany), while *Pae. quisquiliarum* strain SK 55 (JCM 14041) was ordered from Riken BRC, Microbe Division (JCM) (Japan). Strains were revitalized in BHI broth and their purity was verified on BHI agar (Oxoid, UK) streaked plates.

### Digital dDDH and ANI

The reference draft genomes of *Pae. quisquiliarum* strain SK 55 (GCA_900109875) and *Psy. psychrodurans* DSM 11713 (GCA_900114885) were used for genome comparison with the newly sequenced strains, as well as *Psy. psychrodurans* Aquil_B6 (GCA_022603175) already deposited on NCBI database in the previous work. dDDH calculation was performed using the TYGS tool provided by the Leibniz DSMZ Institute (9), while ANI values were calculated using FastANI (35) both used with default settings. A further comparison of the strains was done via JSpeciesWS database, using blastn (ANIb) and the Tetra Correlation Search function (15).

### DNA extraction and genome sequencing

The DNA for genome sequencing was extracted from a fresh cell culture growth overnight at 30°C in BHI broth. Cells were pelleted by centrifugation at 5,000 × g for 10 minutes. The DNA was extracted using the MagAttract HMW DNA kit (Qiagen, Germany) following the manufacturer’s instructions. For genome sequencing, DNA library preparation was performed using the Nextera XT DNA sample preparation kit (Illumina, San Diego, CA, USA) according to the manufacturer’s instructions. First, 1 ng of input DNA from each sample was used for the library preparation which underwent fragmentation by sonication (BioRuptor Diagenode, Belgium), adapter ligation, and amplification (Celero DNA-Seq kit, Tecan, Switzerland). DNA sequencing was performed on a MiSeq platform (Illumina) using a paired-end 250 bp output.

### Genome assembly

The raw reads obtained from the sequencing process were carefully processed with the WGA-LP pipeline (36) using the following tools in default mode. Illumina adapters and quality trims were made with Trimmomatic v0.39 (37). FastQC v0.11.9 (38) was used to assess the quality of trimmed reads, and Kraken2 v2.0.8-b (39) was used to assess the possible presence of contaminants. Assembly was carried out using SPAdes v3.15.2 (40). The quality and completeness of the final assemblies were evaluated using CheckM v1.1.3 (41), Quast v5.0.2 (42), and SamTools v1.10 (43).

### Genome annotation and characterization

The genomes were functionally annotated using Prokka 1.14.6 (44), reconstructing metabolisms and assigning COG annotations to the identified proteins using EggNog (45), and classifying them according to the KEGG mapper function (46). Roary (47) was used in combination with BRIG to generate a BLASTN-based ring map for the analysis of gene distribution across genomes and the computation of the pangenome (48). PHASTER (49), CRISPRCasFinder (50), BAGEL4 (51), and RGI from CARDS (52) were also used to look for the presence of prophages, CRISPR-Cas systems, bacteriocins, and resistance factors. The BacMet database (53) was employed to identify antibacterial biocide and metal resistance genes. The estimation of evolutionary distances was made through phylonium (54).

## Data Availability

All high-throughput sequences obtained for this work are available on NCBI bioprojects PRJNA840842 and PRJNA811801. The corresponding WGS and SRA are available for each strain with these accession numbers: DSM11713 (WGS: JAMKBK000000000; SRA: SRR19330377), DSM30747 (WGS: JAMKBI000000000; SRA: SRR19330375), Aquil_B6 (WGS: JAKXDZ000000000; SRA: SRR18190499), and SK55 (WGS: JAMKBJ000000000; SRA: SRR19330376).

## SUPPLEMENTAL MATERIAL

Table S1 spectrum.00686-23-s0001.pdf KEGG Mapper metabolisms.

ASM does not own the copyrights to Supplemental Material that may be linked to, or accessed through, an article. The authors have granted ASM a non-exclusive, world-wide license to publish the Supplemental Material files. Please contact the corresponding author directly for reuse.
