# Erratum for Colautti et al., “Ancient Roman bacterium against current issues: strain Aquil_B6, *Paenisporosarcina quisquiliarum*, or *Psychrobacillus psychrodurans*?”

**DOI:** 10.1128/spectrum.00405-24

**Published:** 2024-05-23

**Authors:** Andrea Colautti, Giuseppe Comi, Enrico Peterlunger, Lucilla Iacumin

## ERRATUM

Volume 11, no. 6, e00686-23, 2023, https://doi.org/10.1128/spectrum.00686-23. Page 15: Reference 36 should appear as follows.

Rossi N, Colautti A, Iacumin L, Piazza C. 2022. WGA-LP: a pipeline for whole genome assembly of contaminated reads. Bioinformatics 38:846−848. https://doi.org/10.1093/bioinformatics/btab719.

