# Supplementary material for: Ancient Roman bacterium against current issues: strain Aquil_B6, Paenisporosarcina quisquiliarum, or Psychrobacillus psychrodurans?
Source: Microbiol Spectr. 2023 Nov 17;11(6):e00686-23. doi: 10.1128/spectrum.00686-23 (PMC10714998; doi:10.1128/spectrum.00686-23)
Supplement: Table S1 — KEGG Mapper metabolisms. [file spectrum.00686-23-s0001.pdf]

Supplementary Table 1, KEGG Mapper metabolisms

Aquil_B6	DSM 30747	DSM 11713	SK55	KEGG modules	Metabolism
					**Carbohydrate metabolism**
					Central carbohydrate metabolism
green	green	green	green	M00001	Glycolysis (Embden-Meyerhof pathway), glucose => pyruvate
green	green	green	green	M00002	Glycolysis, core module involving three-carbon compounds
green	green	green	green	M00003	Gluconeogenesis, oxaloacetate => fructose-6P
green	green	green	green	M00307	Pyruvate oxidation, pyruvate => acetyl-CoA
green	green	green	green	M00009	Citrate cycle (TCA cycle, Krebs cycle)
green	green	green	green	M00010	Citrate cycle, first carbon oxidation, oxaloacetate => 2-oxoglutarate
green	green	green	green	M00011	Citrate cycle, second carbon oxidation, 2-oxoglutarate => oxaloacetate
orange	orange	orange	orange	M00004	Pentose phosphate pathway (Pentose phosphate cycle)
red	red	red	yellow	M00006	Pentose phosphate pathway, oxidative phase, glucose 6P => ribulose 5P
green	green	green	green	M00007	Pentose phosphate pathway, non-oxidative phase, fructose 6P => ribose 5P
red	red	red	yellow	M00580	Pentose phosphate pathway, archaea, fructose 6P => ribose 5P
green	green	green	green	M00005	PRPP biosynthesis, ribose 5P => PRPP
orange	orange	orange	orange	M00008	Entner-Doudoroff pathway, glucose-6P => glyceraldehyde-3P + pyruvate
yellow	yellow	yellow	yellow	M00308	Semi-phosphorylative Entner-Doudoroff pathway, gluconate => glycerate-3P
					Other carbohydrate metabolism
orange	orange	orange	orange	M00014	Glucuronate pathway (uronate pathway)
orange	orange	orange	orange	M00631	D-Galacturonate degradation (bacteria), D-galacturonate => pyruvate + D-glyceraldehyde 3P
orange	orange	orange	orange	M00061	D-Glucuronate degradation, D-glucuronate => pyruvate + D-glyceraldehyde 3P
green	green	orange	red	M00632	Galactose degradation, Leloir pathway, galactose => alpha-D-glucose-1P
orange	orange	orange	orange	M00552	D-galactonate degradation, De Ley-Doudoroff pathway, D-galactonate => glycerate-3P
orange	orange	orange	orange	M00129	Ascorbate biosynthesis, animals, glucose-1P => ascorbate
orange	orange	orange	orange	M00550	Ascorbate degradation, ascorbate => D-xylulose-5P
green	green	green	red	M00854	Glycogen biosynthesis, glucose-1P => glycogen/starch
orange	orange	orange	orange	M00855	Glycogen degradation, glycogen => glucose-6P
orange	orange	orange	red	M00565	Trehalose biosynthesis, D-glucose 1P => trehalose
green	green	green	green	M00549	Nucleotide sugar biosynthesis, glucose => UDP-glucose
green	green	red	red	M00554	Nucleotide sugar biosynthesis, galactose => UDP-galactose
orange	orange	orange	orange	M00892	UDP-N-acetyl-D-glucosamine biosynthesis, eukaryotes, glucose => UDP-GlcNAc
green	green	green	green	M00909	UDP-N-acetyl-D-glucosamine biosynthesis, prokaryotes, glucose => UDP-GlcNAc
green	green	green	green	M00012	Glyoxylate cycle
orange	orange	orange	orange	M00373	Ethylmalonyl pathway
orange	orange	orange	orange	M00740	Methylaspartate cycle
orange	orange	orange	orange	M00532	Photorespiration
orange	orange	orange	orange	M00013	Malonate semialdehyde pathway, propanoyl-CoA => acetyl-CoA
yellow	yellow	yellow	yellow	M00741	Propanoyl-CoA metabolism, propanoyl-CoA => succinyl-CoA
orange	orange	orange	orange	M00131	Inositol phosphate metabolism, Ins(1,3,4,5)P4 => Ins(1,3,4)P3 => myo-inositol
					**Energy metabolism**
					Carbon fixation
orange	orange	orange	orange	M00165	Reductive pentose phosphate cycle (Calvin cycle)
orange	orange	orange	orange	M00166	Reductive pentose phosphate cycle, ribulose-5P => glyceraldehyde-3P
yellow	yellow	yellow	yellow	M00167	Reductive pentose phosphate cycle, glyceraldehyde-3P => ribulose-5P
yellow	yellow	yellow	yellow	M00168	CAM (Crassulacean acid metabolism), dark
orange	orange	orange	orange	M00170	C4-dicarboxylic acid cycle, phosphoenolpyruvate carboxykinase type
orange	orange	orange	orange	M00173	Reductive citrate cycle (Arnon-Buchanan cycle)
orange	orange	orange	orange	M00376	3-Hydroxypropionate bi-cycle
orange	orange	orange	orange	M00375	Hydroxypropionate-hydroxybutylate cycle
orange	orange	orange	orange	M00374	Dicarboxylate-hydroxybutyrate cycle
orange	orange	orange	orange	M00377	Reductive acetyl-CoA pathway (Wood-Ljungdahl pathway)
green	green	green	green	M00579	Phosphate acetyltransferase-acetate kinase pathway, acetyl-CoA => acetate

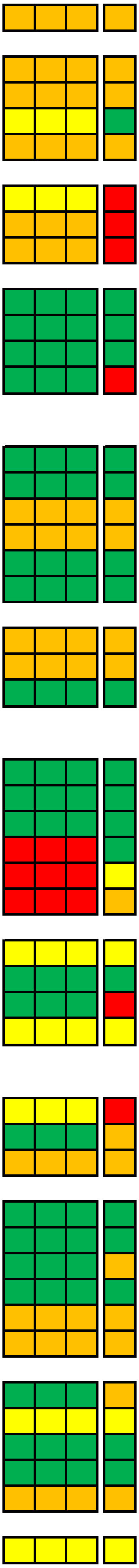

M00620	Incomplete reductive citrate cycle, acetyl-CoA => oxoglutarate
	Methane metabolism
M00357	Methanogenesis, acetate => methane
M00346	Formaldehyde assimilation, serine pathway
M00345	Formaldehyde assimilation, ribulose monophosphate pathway
M00344	Formaldehyde assimilation, xylulose monophosphate pathway
	Nitrogen metabolism
M00530	Dissimilatory nitrate reduction, nitrate => ammonia
M00529	Denitrification, nitrate => nitrogen
M00804	Complete nitrification, comammox, ammonia => nitrite => nitrate
	ATP synthesis
M00151	Cytochrome bc1 complex respiratory unit
M00155	Cytochrome c oxidase, prokaryotes
M00416	Cytochrome aa3-600 menaquinol oxidase
M00157	F-type ATPase, prokaryotes and chloroplasts
	**Lipid metabolism**
	Fatty acid metabolism
M00082	Fatty acid biosynthesis, initiation
M00083	Fatty acid biosynthesis, elongation
M00873	Fatty acid biosynthesis in mitochondria, animals
M00874	Fatty acid biosynthesis in mitochondria, fungi
M00086	beta-Oxidation, acyl-CoA synthesis
M00087	beta-Oxidation
	Lipid metabolism
M00088	Ketone body biosynthesis, acetyl-CoA => acetoacetate/3-hydroxybutyrate/acetone
M00089	Triacylglycerol biosynthesis
M00093	Phosphatidylethanolamine (PE) biosynthesis, PA => PS => PE
	**Nucleotide metabolism**
	Purine metabolism
M00048	Inosine monophosphate biosynthesis, PRPP + glutamine => IMP
M00049	Adenine ribonucleotide biosynthesis, IMP => ADP,ATP
M00050	Guanine ribonucleotide biosynthesis, IMP => GDP,GTP
M00053	Deoxyribonucleotide biosynthesis
M00958	Adenine ribonucleotide degradation, AMP => Urate
M00959	Guanine ribonucleotide degradation, GMP => Urate
	Pyrimidine metabolism
M00051	Uridine monophosphate biosynthesis, glutamine (+ PRPP) => UMP
M00052	Pyrimidine ribonucleotide biosynthesis, UMP => UDP/UTP,CDP/CTP
M00053	Pyrimidine deoxyribonuleotide biosynthesis, CDP => dCTP
M00938	Pyrimidine deoxyribonuleotide biosynthesis, UDP => dTTP
	**Amino acid metabolism**
	Serine and threonine metabolism
M00020	Serine biosynthesis, glycerate-3P => serine
M00018	Threonine biosynthesis, aspartate => homoserine => threonine
M00033	Ectoine biosynthesis, aspartate => ectoine
	Cysteine and methionine metabolism
M00021	Cysteine biosynthesis, serine => cysteine
M00609	Cysteine biosynthesis, methionine => cysteine
M00017	Methionine biosynthesis, apartate => homoserine => methionine
M00034	Methionine salvage pathway
M00035	Methionine degradation
M00368	Ethylene biosynthesis, methionine => ethylene
	Branched-chain amino acid metabolism
M00019	Valine/isoleucine biosynthesis, pyruvate => valine / 2-oxobutanoate => isoleucine
M00535	Isoleucine biosynthesis, pyruvate => 2-oxobutanoate
M00570	Isoleucine biosynthesis, threonine => 2-oxobutanoate => isoleucine
M00432	Leucine biosynthesis, 2-oxoisovalerate => 2-oxoisocaproate
M00036	Leucine degradation, leucine => acetoacetate + acetyl-CoA
	Lysine metabolism
M00016	Lysine biosynthesis, succinyl-DAP pathway, aspartate => lysine

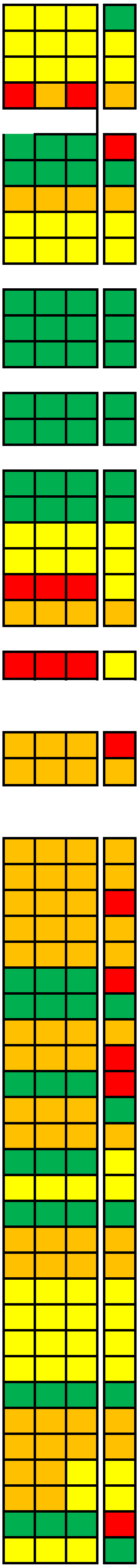

M00525 Lysine biosynthesis, acetyl-DAP pathway, aspartate => lysine
M00526 Lysine biosynthesis, DAP dehydrogenase pathway, aspartate => lysine
M00527 Lysine biosynthesis, DAP aminotransferase pathway, aspartate => lysine
M00030 Lysine biosynthesis, AAA pathway, 2-oxoglutarate => 2-aminoadipate => lysine

Arginine and proline metabolism

M00028 Ornithine biosynthesis, glutamate => ornithine
M00844 Arginine biosynthesis, ornithine => arginine
M00845 Arginine biosynthesis, glutamate => acetylcitrulline => arginine
M00029 Urea cycle
M00015 Proline biosynthesis, glutamate => proline

Polyamine biosynthesis

M00133 Polyamine biosynthesis, arginine => agmatine => putrescine => spermidine
M00134 Polyamine biosynthesis, arginine => ornithine => putrescine
M00135 GABA biosynthesis, eukaryotes, putrescine => GABA

Histidine metabolism

M00026 Histidine biosynthesis, PRPP => histidine
M00045 Histidine degradation, histidine => N-formiminoglutamate => glutamate

Aromatic amino acid metabolism

M00022 Shikimate pathway, phosphoenolpyruvate + erythrose-4P => chorismate
M00023 Tryptophan biosynthesis, chorismate => tryptophan
M00024 Phenylalanine biosynthesis, chorismate => phenylpyruvate => phenylalanine
M00025 Tyrosine biosynthesis, chorismate => HPP => tyrosine
M00533 Homoprotocatechuate degradation, homoprotocatechuate => 2-oxohept-3-enedioate
M00038 Tryptophan metabolism, tryptophan => kynurenine => 2-aminomuconate

Other amino acid metabolism

M00027 GABA (gamma-Aminobutyrate) shunt

Glycan metabolism

Lipopolysaccharide metabolism

M00064 ADP-L-glycero-D-manno-heptose biosynthesis
M00922 CMP-Neu5Ac biosynthesis

Metabolism of cofactors and vitamins

Cofactor and vitamin metabolism

M00127 Thiamine biosynthesis, prokaryotes, AIR (+ DXP/tyrosine) => TMP/TPP
M00895 Thiamine biosynthesis, prokaryotes, AIR (+ DXP/glycine) => TMP/TPP
M00896 Thiamine biosynthesis, archaea, AIR (+ NAD+) => TMP/TPP
M00897 Thiamine biosynthesis, plants, AIR (+ NAD+) => TMP/thiamine/TPP
M00898 Thiamine biosynthesis, pyridoxal-5P => TMP/thiamine/TPP
M00899 Thiamine salvage pathway, HMP/HET => TMP
M00125 Riboflavin biosynthesis, plants and bacteria, GTP => riboflavin/FMN/FAD
M00911 Riboflavin biosynthesis, fungi, GTP => riboflavin/FMN/FAD
M00124 Pyridoxal-P biosynthesis, erythrose-4P => pyridoxal-P
M00916 Pyridoxal-P biosynthesis, R5P + glyceraldehyde-3P + glutamine => pyridoxal-P
M00115 NAD biosynthesis, aspartate => quinolinate => NAD
M00912 NAD biosynthesis, tryptophan => quinolinate => NAD
M00119 Pantothenate biosynthesis, valine/L-aspartate => pantothenate
M00913 Pantothenate biosynthesis, 2-oxoisovalerate/spermine => pantothenate
M00120 Coenzyme A biosynthesis, pantothenate => CoA
M00914 Coenzyme A biosynthesis, archaea, 2-oxoisovalerate => 4-phosphopantoate => CoA
M00572 Pimeloyl-ACP biosynthesis, BioC-BioH pathway, malonyl-ACP => pimeloyl-ACP
M00881 Lipoic acid biosynthesis, plants and bacteria, octanoyl-ACP => dihydrolipoyl-E2/H
M00882 Lipoic acid biosynthesis, eukaryotes, octanoyl-ACP => dihydrolipoyl-H
M00883 Lipoic acid biosynthesis, animals and bacteria, octanoyl-ACP => dihydrolipoyl-H => dihydrolipoyl-E2
M00884 Lipoic acid biosynthesis, octanoyl-CoA => dihydrolipoyl-E2
M00126 Tetrahydrofolate biosynthesis, GTP => THF
M00840 Tetrahydrofolate biosynthesis, mediated by ribA and trpF, GTP => THF
M00841 Tetrahydrofolate biosynthesis, mediated by PTPS, GTP => THF
M00842 Tetrahydrobiopterin biosynthesis, GTP => BH4
M00843 L-threo-Tetrahydrobiopterin biosynthesis, GTP => L-threo-BH4
M00880 Molybdenum cofactor biosynthesis, GTP => molybdenum cofactor
M00140 C1-unit interconversion, prokaryotes

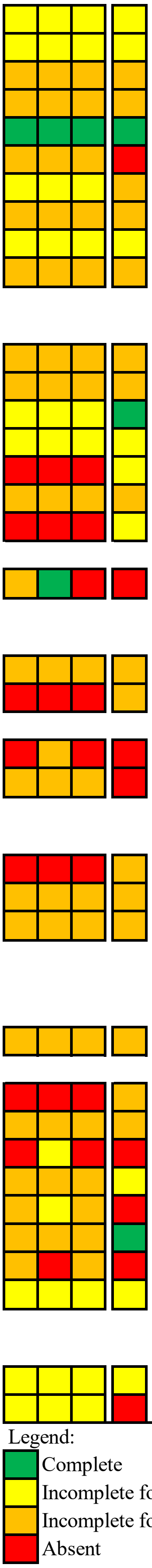

M00141 C1-unit interconversion, eukaryotes
M00846 Siroheme biosynthesis, glutamyl-tRNA => siroheme
M00868 Heme biosynthesis, animals and fungi, glycine => heme
M00121 Heme biosynthesis, plants and bacteria, glutamate => heme
M00926 Heme biosynthesis, bacteria, glutamyl-tRNA => coproporphyrin III => heme
M00847 Heme biosynthesis, archaea, siroheme => heme
M00122 Cobalamin biosynthesis, cobyrinate a,c-diamide => cobalamin
M00117 Ubiquinone biosynthesis, prokaryotes, chorismate (+ polyprenyl-PP) => ubiquinol
M00116 Menaquinone biosynthesis, chorismate (+ polyprenyl-PP) => menaquinol
M00932 Phylloquinone biosynthesis, chorismate (+ phytyl-PP) => phylloquinol

Biosynthesis of terpenoids and polyketides

Terpenoid backbone biosynthesis

M00095 C5 isoprenoid biosynthesis, mevalonate pathway
M00849 C5 isoprenoid biosynthesis, mevalonate pathway, archaea
M00096 C5 isoprenoid biosynthesis, non-mevalonate pathway
M00364 C10-C20 isoprenoid biosynthesis, bacteria
M00365 C10-C20 isoprenoid biosynthesis, archaea
M00366 C10-C20 isoprenoid biosynthesis, plants
M00367 C10-C20 isoprenoid biosynthesis, non-plant eukaryotes

Polyketide sugar unit biosynthesis

M00793 dTDP-L-rhamnose biosynthesis

Biosynthesis of other secondary metabolites

Biosynthesis of phytochemical compounds

M00039 Monolignol biosynthesis, phenylalanine/tyrosine => monolignol
M00942 Pterocarpan biosynthesis, daidzein => medicarpin

Biosynthesis of other antibiotics

M00877 Kanosamine biosynthesis glucose 6-phosphate => kanosamine
M00787 Bacilysin biosynthesis, prephenate => bacilysin

Xenobiotics biodegradation

Aromatics degradation

M00568 Catechol ortho-cleavage, catechol => 3-oxoadipate
M00569 Catechol meta-cleavage, catechol => acetyl-CoA / 4-methylcatechol => propanoyl-CoA
M00878 Phenylacetate degradation, phenylaxetate => acetyl-CoA/succinyl-CoA

Signature modules

Gene set

Pathogenicity

M00860 Bacillus anthracis pathogenicity signature, polyglutamic acid capsule biosynthesis

Drug resistance

M00625 Methicillin resistance
M00627 beta-Lactam resistance, Bla system
M00704 Tetracycline resistance, efflux pump Tet38
M00725 Cationic antimicrobial peptide (CAMP) resistance, dltABCD operon
M00726 Cationic antimicrobial peptide (CAMP) resistance, lysyl-phosphatidylglycerol (L-PG) synthase MprF
M00730 Cationic antimicrobial peptide (CAMP) resistance, VraFG transporter
M00769 Multidrug resistance, efflux pump MexPQ-OpmE
M00700 Multidrug resistance, efflux pump AbcA

Module set

Metabolic capacity

M00618 Acetogen
M00615 Nitrate assimilation

Legend:
Complete
Incomplete for 1 step
Incomplete for >1 step
Absent
